# UCNP-based Photoluminescent Nanomedicines for Targeted Imaging and Theranostics of Cancer

**DOI:** 10.3390/molecules25184302

**Published:** 2020-09-19

**Authors:** Evgenii L. Guryev, Anita S. Smyshlyaeva, Natalia Y. Shilyagina, Evgeniya A. Sokolova, Samah Shanwar, Alexey B. Kostyuk, Alexander V. Lyubeshkin, Alexey A. Schulga, Elena V. Konovalova, Quan Lin, Indrajit Roy, Irina V. Balalaeva, Sergey M. Deyev, Andrei V. Zvyagin

**Affiliations:** 1 Institute of Biology and Biomedicine, Lobachevsky State University of Nizhny Novgorod, 603950 Nizhny Novgorod, Russia; eguryev@ibbm.unn.ru (E.L.G.); smysh.anita@gmail.com (A.S.S.); nat-lekanova@yandex.ru (N.Y.S.); malehanova@mail.ru (E.A.S.); samahshanwar@googlemail.com (S.S.); kostyukalexey@mail.ru (A.B.K.); andrei.zvyagin@mq.edu.au (A.V.Z.); 2 Federal Scientific Research Center “Crystallography and Photonics”, Russian Academy of Sciences, 119333 Moscow, Russia; cito2006@rambler.ru; 3 Shemyakin-Ovchinnikov Institute of Bioorganic Chemistry, Russian Academy of Sciences, 117997 Moscow, Russia; schulga@gmail.com (A.A.S.); elena.ko.mail@gmail.com (E.V.K.); deyev@ibch.ru (S.M.D.); 4 College of Chemistry, Jilin University, Changchun 130012, China; linquan@jlu.edu.cn; 5 Department of Chemistry, University of Delhi, Delhi 110007, India; iroy@chemistry.du.ac.in; 6 The Institute of Molecular Medicine, I.M. Sechenov First Moscow State Medical University, 119991 Moscow, Russia

**Keywords:** immunoconjugates, tumor, imaging, therapy, drug, antigen

## Abstract

Theranostic approach is currently among the fastest growing trends in cancer treatment. It implies the creation of multifunctional agents for simultaneous precise diagnosis and targeted impact on tumor cells. A new type of theranostic complexes was created based on NaYF_4_: Yb,Tm upconversion nanoparticles coated with polyethylene glycol and functionalized with the HER2-specific recombinant targeted toxin DARPin-LoPE. The obtained agents bind to HER2-overexpressing human breast adenocarcinoma cells and demonstrate selective cytotoxicity against this type of cancer cells. Using fluorescent human breast adenocarcinoma xenograft models, the possibility of intravital visualization of the UCNP-based complexes biodistribution and accumulation in tumor was demonstrated.

## 1. Introduction

A personalized approach is becoming increasingly important in the treatment of oncological diseases. Theranostics, the trend of biomedicine, which implies the creation of supramolecular complexes with diagnostic and therapeutic properties, fully meets its requirements [1,2]. The study of the molecular peculiarities of tumors and the development of nanotechnology have made it possible to create a number of promising agents based on nanomaterials and intended for diagnostics and targeted therapy [3,4,5].

Photoluminescent nanomaterials are objects of great interest for the design of theranostic agents. The most promising of them seem to be upconversion nanoparticles (UCNP)–inorganic crystals containing ions of trivalent lanthanides, capable of converting low-energy radiation into radiation with a shorter wavelength and higher energy [6]. UCNP emits photoluminescence (PL) in the visible and infrared (IR) regions under IR light excitation, and this reduces the absorption and scattering of the excitation radiation and allows one to clearly separate the target photoluminescence signal from tissue autofluorescence [7]. The combination of photophysical and chemical properties of UCNP makes them highly demanded agents for intravital labeling of various targets, including tumor cells [8,9,10,11]. The possibility of attaching additional targeting and therapeutic components to UCNP surface makes it possible to obtain multifunctional agents for a variety of theranostic applications [5,12,13].

Due to synthesis procedure, UCNP carry hydrophobic oleate groups on surface, which makes them unstable in aqueous solutions and non-biocompatible. Coating with amphiphilic polymers is an effective way to hydrophilizing UCNP surface, which can also form functional groups capable of attaching various biomolecules. Targeted delivery strategies for such complexes are rather diverse. Selective accumulation in the tumor tissue is an essential factor due to the enhanced permeability and retention (EPR) effect [14,15], caused by the tumor’s abnormal vasculature. The vascular bed of the tumor differs in structure and function from normal tissues, which allows large molecules and nanoparticles to penetrate into the tumor tissue and retain in it. Macrophages located in the tumor are also able to accumulate such substances, thereby increasing their retention time [16]. Despite the fact that a number of anticancer carrier-drug systems are successfully used in the clinic, the development of approaches combining the use of the EPR effect with other methods of directed delivery remains to be of high relevance [17,18]. The efficient targeted delivery of nanocomplexes to the tumor site requires important features, which are the colloidal stability and the ability to circulate for a long time in the bloodstream. These parameters can be provided by polyethylene glycol coating of UCNP. Despite some shortcomings, PEGylation is one of the most important strategies for nanoparticles surface modification, since it remarkably increases its blood circulation time, prevents enzymatic degradation, opsonization, macrophage uptake, and accumulation in the liver [19].

The general approach for increasing the selectivity of intracellular accumulation and retention time, called ‘active targeting’, is to use protein targeting modules capable of selective binding to a particular type of receptors overexpressed on tumor cells surface [20,21]. Active targeting acts when the targeting agent and the target interact directly at distances not exceeding 0.5 nm [22]. A wide range of proteins are currently used as molecular targets, in particular, epidermal growth factor receptors EGFR (HER1–4) [23], vascular endothelial growth factor receptors VEGFR 1-3 and integrins [24], platelet growth factor receptors PDGFRα/β [25], insulin-like growth factor receptor IGF-1R [26], interleukins receptors, folic acid receptors [27], vitamins receptors [28] and others. One of the most significant tumor markers is HER2, a human epidermal growth factor receptor, overexpression is directly related to the growth of various types of malignant tumors (breast and cervix adenocarcinoma, ovary and lung carcinoma, etc.) [20] and indicates their aggressiveness and high metastatic potential [29,30,31]. Therefore, the creation of HER2-specific complexes is an actual direction in diagnostics and therapy.

Full-length antibodies or their derivatives are widely used as targeting molecules: antibody fragments, single-chain antibody fragments-scFv (single chain fragment variable), dsFv (disulfide-stabilized fragment variable), and sdAb (single-domain antibody, nanobody) [32]. A promising alternative to antibodies is non-immunoglobulin scaffolds. They also contain hypervariable regions that allow highly specific target recognition [33]. If the membrane receptors are used as molecular targets, a therapeutic agent can be attached to a targeting molecule, which is used in the construction of anticancer drugs.

DARPins (Designed Ankyrin Repeat Proteins) are widely used as targeting modules due to their small molecule size, high stability in various conditions, high-yield production in heterologous expression systems, and efficient folding. DARPins consist of several ankyrin repeats (4–6) forming a right-handed solenoid, which has an extended hydrophobic core and a hydrophilic outer surface [34]. DARPin9.29 (hereinafter DARPin) used in this study was obtained in the laboratory of A. Plückthun. This protein is able to interact with the extracellular domain of the HER2 receptor with high affinity, comparable to or even superior to that of scFv antibodies (*K*_D_ = 3.8 nM) [35]. The advantages of DARPins over immunoglobulins allow us to expect their effective use as targeted modules for the creation of multifunctional agents.

For the development of a personalized approach, it is important to be able to choose a treatment strategy and a drug delivery method depending on the morphological and molecular characteristics of the tumor. Various toxic modules are used for a therapeutic effect on tumor cells: chemotherapy and radionuclide therapy drugs, photosensitizers, protein toxins [5,36,37]. Among the latter, the most interesting are bifunctional target toxins capable of acting both as targeting molecules and as toxic agents [38,39]. Exotoxin A from *Pseudomonas aeruginosa* (PE) is one of the most effective protein toxins. It consists of three domains responsible for binding and internalization into the animal cells, intracellular processing, and catalytic activity leading to toxic effect through ADP-ribosylation of eukaryotic elongation factor 2 and blocking the protein synthesis. The risk of immunogenicity and general toxicity for all anticancer agents of a protein nature may prohibit repetition of treatment courses. This risk can be reduced by using of an effector module with low immunogenicity such as LoPE–fragment of *Pseudomonas* exotoxin A in which the recognition sites of human B cells are removed or inactivated [40]. LoPE lacks its own binding domain and attached to the C-terminus of DARPin through a flexible hydrophilic 16-amino acid linker.

Recently created targeted toxin DARPin-LoPE [41] consists of a HER2-specific DARPin and a low immunogenic LoPE and can act as a targeting and toxic module in nanocomplexes. DARPin-LoPE exhibits high activity against HER2-overexpressing cancer cells both in vitro and in vivo by inducing apoptosis [40,42]. It was also shown that DARPin-LoPE effectively inhibits the growth of HER2-expressing human ovarian carcinoma xenografts and exhibits low immunogenicity and general toxicity which limit the use of PE-based toxins: less pronounced side effects, specifically vascular leak syndrome, liver and kidney tissue degradation [42]. It also leads to formation of the lower antibody titer following the treatment course, and rapid decline in concentration of specific antibodies.

In this study, we obtained a new type of theranostic nanocomplexes based on UCNP coated with an amphiphilic polymer and heterobifunctional polyethylene glycol (PEG) derivative. Molecules of the recombinant targeted toxin DARPin-LoPE, specific to the clinically significant tumor marker HER2, were attached to the PEG functional groups. Selective labelling and specific toxicity on human breast adenocarcinoma cells overexpressing the HER2 receptor with obtained UCNP-DARPin-LoPE nanocomplexes was shown.

## 2. Results

### 2.1. Preparation and Characterization of Theranostic Nanocomplexes

In the present study two types of theranostic nanocomplexes were obtained: UCNP coated with PMAO-PEG (I) for imaging tumors using passive delivery due to EPR-effect, and nanocomplexes based on UCNP and the target toxin DARPin-LoPE (II) for specific imaging and targeted therapeutic effects on HER2-positive tumor cells.

**Figure 1:**
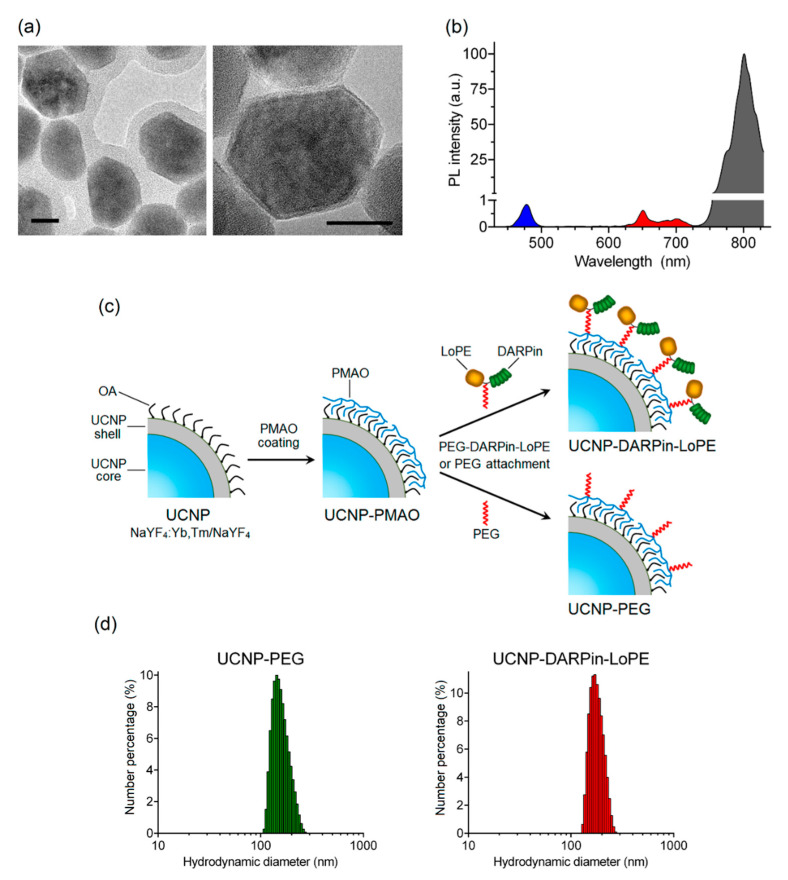
Preparation and characterization of theranostic nanocomplexes. TEM images of upconversion nanoparticles (UCNP) (NaYF_4_: Yb, Tm/NaYF_4_). Scale bar 20 nm. (**a**). PL emission spectrum of UCNP under excitation at 980 nm (**b**). The preparation scheme of UCNP-DARPin-LoPE nanocomplexes and non-targeted UCNP-PEG (**c**). Hydrodynamic diameter distributions of UCNP- polyethylene glycol (UCNP-PEG) (left) and (**d**) UCNP-DARPin-LoPE (right) acquired by dynamic light scattering in aqueous solutions.

The nanocomplexes were based on the UCNP with a core/shell structure NaY_0.794_Yb_0.2_Tm_0.006_F_4_/NaYF_4_ synthesized by the solvothermal decomposition method, which makes it possible to obtain UCNP with desired size and shape. The method includes coordinate stabilization of lanthanide precursors and fluorine precursors in a solution of oleic acid when heated in an oxygen-free environment. As a result, a nanodispersed powder of UCNP crystals with a cubic (α-phase) crystal lattice was obtained, and after additional heat treatment, they were transferred to a more stable β-hexagonal phase. The transition of crystals from α-phase to β-phase was confirmed using X-ray diffraction (Figure S1). The transmission electron microscopy image (Figure 1a) shows the obtained UCNP with an average diameter of 43.8 ± 3.9 nm, predominantly of a hexagonal shape. The inert shell of the UCNP is not visible in micrographs, since the materials of the core and shell of the UCNP have the same electron absorption indices.

Trivalent lanthanide ions ytterbium (Yb^3+^) and thulium (Tm^3+^) were used for doping the NaYF_4_ matrix. As a result, UCNP with PL emission maxima in the blue region (at a wavelength of 474 nm) and in the IR region (at a wavelength of 801 nm) were obtained (Figure 1b). The highest PL intensity in the IR region of the spectrum promotes the application of these UCNP for efficient bioimaging and allows one to record the target PL signal with high sensitivity through a biological tissue layer up to a centimeter deep.

To achieve hydrophilicity UCNP was coated with a layer of amphiphilic alternating copolymer of maleic anhydride with 1-octadecene (PMAO) (Figure 1c) and the hydrodynamic diameter of resulting UCNP-PMAO was 146 ± 20 nm (Table 1). The targeting function and therapeutic effect of nanocomplexes in the present study was realized by conjugation of a PEG-modified targeted toxin DARPin-LoPE to UCNP (Figure 1c). Thus, the combination of the properties of UCNP and targeted toxin DARPin-LoPE allow us to create nanocomplexes for visualization and treatment of HER2-overexpressing tumors.

A heterobifunctional polyethylene glycol derivative (SH-PEG-NH2) was attached to the amino-functional groups of the target toxin DARPin-LoPE using an SMPB linker containing reactive NHS ester and maleimide groups at opposite ends of a medium-length aromatic spacer (11.6 angstroms). The PEG-modified protein DARPin-LoPE was conjugated with UCNP-PMAO using zero-length EDC and sulfo-NHS linkers [43]. In the course of the reaction, EDC interacts with the carboxyl group of the PMAO, forming an intermediate in the presence of sulfo-NHS that actively reacts with the amino group. In the case of non-targeted UCNP-PEG particles, SH-PEG-NH_2_ was attached to UCNP-PMAO in the same way. As a result of the conjugation, UCNP-PEG and UCNP–DARPin-LoPE complexes were obtained with an average particle diameter of 165 ± 35 nm and 179 ± 27 nm respectively (Figure 1d, Table 1). Histograms of particle hydrodynamic diameter distributions by number of UCNP-PEG and UCNP–DARPin-LoPE acquired by dynamic light scattering in aqueous solutions are shown in Figure 1d. A significant increase in size relative to the initial "dry" UCNP is caused by the formation of a hydration shell around the layer of amphiphilic polymer PMAO and, in the case of UCNP–DARPin-LoPE nanocomplexes, attached targeted toxin molecules. Besides, nanoparticle aggregates skewed the distribution towards the larger mean diameter of the ensemble of colloidal nanoparticles. The values of the polydispersity index (Table 1) make it possible to consider the UCNP-PEG and UCNP–DARPin-LoPE particles rather uniform in size. Attachment of functional proteins (DARPin-LoPE) slightly increased the mean hydrodynamic diameter of the colloid and indicated successful conjugation. Successful assembly of nanocomplexes was confirmed by the FTIR spectroscopy (Figure S2). Additionally, the DARPin-LoPE attachment was proved by BCA method. The DARPin-LoPE protein concentration in the complexes was 38 μg/mL at a UCNP concentration of 1 mg/mL in the suspension. The amount of protein per 1 particle was ~150 molecules. Hydrodynamic diameter and the ζ-potential values of UCNP-PEG UCNP-DARPin-LoPE suggest their efficient delivery to the tumor via the passive pathway.

### 2.2. Analysis of the Effectiveness of UCNP-DARPin-LoPE Nanocomplexes In Vitro

A culture of human breast adenocarcinoma cells (SKBR-kat), overexpressing the HER2 receptor on the surface, was used to study the binding specificity of the nanocomplexes UCNP–DARPin-LoPE. Chinese hamster ovary cells (CHO) were used as control (no HER2 expression). In order to prevent internalization, the cells were incubated in the presence of UCNP-PEG/UCNP-DARPin-LoPE at 4 °C for 1 h. The specificity of binding of UCNP-PEG/UCNP-DARPin-LoPE to cells was analyzed by wide-field fluorescence microscopy system, modified for registration of upconversion PL.

**Figure 2:**
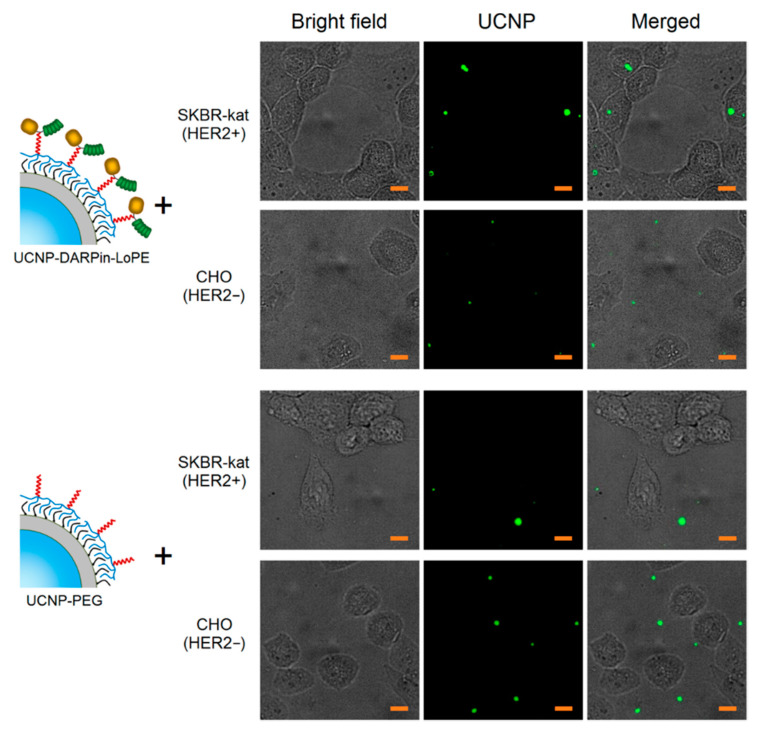
Microscopic images of SKBR-kat and CHO cells incubated with non-targeted UCNP-PEG and UCNP-DARPin-LoPE theranostic nanocomplexes. UCNP–PL signal in the range of 420–840 nm, excitation on 980 nm. The scale section is 10 μm.

The micrographs of SK-BR-3 cells after incubation with UCNP-DARPin-LoPE nanocomplexes show selective accumulation of particles on cells (Figure 2). Nanocomplexes are localized on the surface and/or inside cells. In micrographs of CHO cells after incubation with UCNP-DARPin-LoPE nanocomplexes, the accumulation of particles on the cells is much less pronounced; the particles are arranged chaotically and predominantly adsorbed on the surface of the culture plastic. The incubation of cells of both lines with non-targeted UCNP-PEG also does not lead to a significant accumulation of particles. Microscopic data are consistent with the assumption of specific binding of UCNP-DARPin-LoPE nanocomplexes to the surface of cells overexpressing the HER2 receptor.

**Figure 3:**
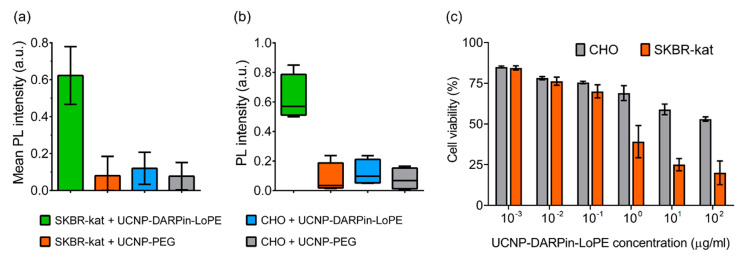
Interaction of non-targeted UCNP-PEG and UCNP-DARPin-LoPE theranostic nanocomplexes with cancer cells. Mean PL intensity of SKBR-kat and CHO cells labeled by UCNP-PEG/UCNP-DARPin-LoPE (**a**), Diagram of the range by groups (**b**). The dependence of the viability of CHO (HER2−) and SKBR-kat (HER2+) cells on the UCNP-DARPin-LoPE nanocomplexes concentration. Incubation time 96 hours (**c**).

The binding specificity of the UCNP-DARPin-LoPE nanocomplexes to the target cells was confirmed by quantitative assessment of the UCNP PL signal in the regions occupied by the cells (Figure 3a). The significance of differences between groups was checked using the nonparametric Kruskal-Wallis H-test (Figure 3b). The data analysis showed a statistically significant difference in the UCNP PL signal intensity on SKBR-kat cells incubated with the UCNP-DARPin-LoPE nanocomplexes from the other variants (*p* = 0.0257 < 0.05). The highest rank sum belongs to group 1 (UCNP-DARPin-LoPE + SKBR-kat), which confirms the initial hypothesis of the experiment (high binding specificity of UCNP-DARPin-LoPE to SKBR-kat cells).

Cytotoxicity study showed that the UCNP-DARPin-LoPE nanocomplexes significantly decreased the viability of SKBR-kat cells overexpressing the HER2 receptor (Figure 3c). The IC_50_ for this cell line was 0.4 μg/mL. No significant cytotoxic effect was observed to HER2-negative CHO cells up to nanocomplexes concentrations of 200 μg/mL. Non-targeted UCNP-PEG at the same concentrations did not affect the viability of both SKBR-kat and CHO cells.

### 2.3. Tumor Model Visualization

The efficient delivery of multifunctional theranostic nanocomplexes to a target organ or tissue, particularly a tumor, is an important aspect of development of such agents. The efficiency of delivery and the dynamics of clearance from the tumor depend on various factors, including the duration of the agent’s circulation in the bloodstream, the development of the tumor vascular and lymphatic network, the targeting properties (specificity to tumor cells) of the complexes themselves, etc. The choice of a suitable tumor model and a method of in vivo imaging are essential for studying the biodistribution of nanocomplexes. Genetic labelling of tumor cells with fluorescent proteins is among the most informative method for studying tumor growth and assessing the effectiveness of tested therapeutic agents.

**Figure 4:**
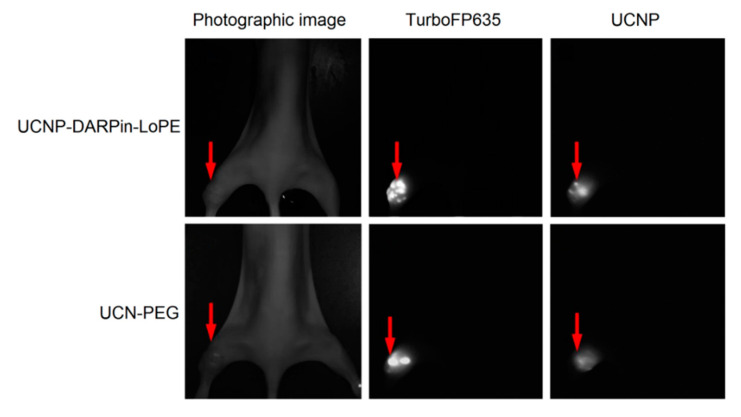
Intravital images of an immunodeficient mouse (BALB/c Nude) with SKBR-kat xenograft tumor 24 h after intratumoral injection of UCN-PEG or UCNP-DARPin-LoPE nanocomplexes. TurboFP635–fluorescent signal of the TurboFP635 protein in the range of 610–700 nm with excitation at 594 nm; UCNP–PL signal of UCNP in the range of 485–831 nm with excitation at 980 nm. The tumor location is indicated by an arrow.

The possibility of simultaneous recording of the fluorescent signal from tumor cells and UCNP PL signal from injected nanocomplexes was demonstrated using the experimental red fluorescent tumor model (Figure 4). Stable fluorescent cell line SKBR-kat was obtained by transfection of human breast adenocarcinoma cells (SKBR-3) with TurboFP635 fluorescent protein [44]. BALB/c Nude immunodeficient mice were subcutaneously inoculated into the thighs with SKBR-kat cells to obtain human breast adenocarcinoma xenografts and UCNP-PEG/UCNP-DARPin-LoPE nanocomplexes were injected intratumorally.

Images based on the registration of Stokes and anti-Stokes PL were obtained using home-built systems for laboratory animal imaging. As can be seen in Figure 4, this approach makes it possible to precisely determine the location of tumor nodes. A quantitative analysis of the UCNP PL signal in the tumor area allows one to assess the selectivity of accumulation and the dynamics of retention of various types of nanocomplexes in the tumor.

## 3. Discussion

Nowadays, nanotheranostics have been approved for use in clinical practice despite being a fairly young direction that is only developing. Several nanoparticle-based theranostic complexes are being used for the treatment of solid tumors, and many others are undergoing various phases of preclinical and clinical trials [45]. Among the variety of nanoparticles, UCNP have drawn the special attention of researchers in recent years with their excitation by low-intensity radiation in the infrared range, which is capable of penetrating most deeply into biological tissues, leading to emission in the visible and IR regions. Another important feature of UCNP is the long lifetime of the excited state, which allows the visualization of labeled targets after the fading of the autofluorescence of biological tissues [7]. The use of UCNP, in relation to their unique photophysical properties, makes it possible to significantly expand the capabilities of traditional methods of optical diagnostics, which are based on the use of organic dyes and fluorescent proteins as labels for visualizing pathological targets [4]. In addition, nanoparticles serve as an excellent platform for the creation of multicomponent theranostic complexes by attaching to them targeted and toxic modules (for example, chemotherapy and radiotherapy agents, protein toxins) to target a specific type of tumors [5,12,13].

In this study, we obtained a new type of theranostic nanocomplexes based on UCNP coated with PEG and functionalized with the recombinant targeted toxin DARPin-LoPE [40,42]. Selective binding of UCNP-DARPin-LoPE nanocomplexes to HER2-overexpressing human breast adenocarcinoma cells was shown. Besides, a significant decrease in cancer cell viability was revealed due to the selective action of the targeted toxin DARPin-LoPE by blocking protein biosynthesis [42] (see also Figure S3). The demonstrated ability of the obtained nanocomplexes to visualize tumors indicates their potential as agents for bioimaging.

The nanocomplexes created in this study can be applied for the diagnosis and therapy of a number of HER2-overexpressing tumors. Due to the modular structure, the complexes can include diagnostic and therapeutic components with different specificity and mechanism of action and it make it possible to obtain theranosic agents with a wide spectrum of properties. For delivery of nanocomplexes to a tumor, both a passive pathway due to the EPR effect and an active mechanism due to targeting modules can be used. The main advantage of passive targeting is that it can be applied to large tumors by systemic intravenous injection. Using the active delivery allows targeting cells overexpressing certain tumor markers. The results of the study indicate the broad possibilities of using the developed photoluminescent nanocomplexes in theranostics of cancer.

## 4. Materials and Methods

### 4.1. Synthesis of UCNP 

Hydrophobic UCNP in the form of NaYF_4_ crystals doped with Yb^3+^ and Tm^3+^ ions and having an oleate anion on the surface were synthesized by the solvothermal decomposition method [46]. A mixture containing Y_2_O_3_ (0.794 mM), Yb_2_O_3_ (0.2 mM), and Tm_2_O_3_ (0.006 mM) was dissolved in 70% trifluoroacetic acid in a three-necked flask, then evaporated. Sodium trifluoroacetate to 2.2 mM, 7.5 mL of oleic acid, and 7.5 mL of 1-octadecene were added into the flask, and mixture was vacuum-dried under heating and purging with argon. The reaction mixture was incubated until turbidity in Wood’s alloy at 343 °C, and then at 312 °C until transparency was restored. The mixture was cooled down to 210 °C, and 4 mL of 1-octadecene were added. UCNP (core) crystals were precipitated with isopropanol, resuspended in hexane, and washed three times with ethanol. In order to form the UCNP shell, 60 mg of sodium trifluoroacetate, 140 mg of yttrium trifluoroacetate, 3 mL of oleic acid and 3 mL of 1-octadecene were added to the resulting suspension, then incubated in Wood’s alloy sequentially at 100 °C, 170 °C, 210 °C and 290 °C. Then 3 mL of 1-octadecene were added to the solution, UCNP (core/shell) was precipitated and washed as described above and resuspended in chloroform.

### 4.2. Characterization of UCNP 

The PL properties of UCNP were analyzed using an SM 2203 spectrofluorimeter (SOLAR, Minsk, Belarus) and an external semiconductor laser module ATC-C4000-200AMF-980-5-F200 with a wavelength of 980 nm (Semiconductor devices, Saint-Petersburg, Russia). The PL emission spectra were measured in the range from 400 to 850 nm in a quartz cell with an optical path length of 1 cm. The ζ-potential was determined, and the hydrodynamic diameter of UCNP and nanocomplexes was measured by electrophoretic light scattering and dynamic light scattering, respectively, using the Zetasizer Nano ZS (Malvern Instruments Ltd., Malvern, UK) according to the manufacturer’s recommendations.

### 4.3. Production of DARPin-LoPE Protein 

*E.coli* BL21(DE3) strain cells were transformed with the pDARP-LoPE plasmid (provided by the laboratory of molecular immunology of Shemyakin-Ovchinnikov Institute of Bioorganic Chemistry, Russian Academy of Sciences, Moscow, Russia). Colonies of transformants were transferred into 1 L of culture medium (1% yeast extract, 1% tryptone, 25 mM Na_2_HPO_4_, 25 mM KH_2_PO_4_, 100 mM NaCl, 2 mM MgCl_2_, 0.1 g/L ampicillin) and cultured at 37 °C under intensive stirring. When the culture reached OD_600_ = 0.5, the temperature was lowered to 28 °C, the expression of the target protein was induced by adding isopropyl thiogalactopyranoside to a final concentration of 1 mM, and the culture was incubated for 8 h with stirring. The cells were harvested by centrifugation and resuspended in a buffer containing 500 mM NaCl, 20 mM Na_2_HPO_4_, 30 mM imidazole, pH 7.5. The target protein was purified by nickel affinity chromatography: the cells were disrupted by ultrasound, the clarified lysate was filtered and loaded onto a HisTrap FF column (GE Healthcare, Piscataway, NJ, USA) equilibrated with a buffer (20 mM Na_2_HPO_4_, 500 mM NaCl, and 30 mM imidazole, pH 7.5). The target DARPin-LoPE protein was eluted with a linear imidazole gradient from 30 to 500 mM. The obtained protein was transferred into phosphate-buffered saline pH 7.4 (PBS) buffer using a PD MidiTraP G-25 column (GE Healthcare, Piscataway, NJ, USA).

### 4.4. Coating of UCNP with a Copolymer of Maleic Anhydride and 1-octadecene (PMAO)

1.04 mg of PMAO dissolved in 130 μL of chloroform was added to 50 μL of UCNP suspension in chloroform (50 mg/mL) and sonicated for 30 s. Mixture was incubated with stirring until 4/5 of the volume evaporated. Mixture was added dropwise in a tube with 1 mL of PBS with stirring and sonication. The suspension was sonicated for 30 min until the chloroform had completely evaporated and washed three times with PBS.

### 4.5. Conjugation of DARPin-LoPE Protein with Heterobifunctional SH-PEG-NH_2_

A 30-fold molar excess of N-succinimidyl-4-(p-maleimidophenyl)-butyrate (SMPB) in dimethyl sulfoxide (DMSO) was added to a DARPin-LoPE solution in PBS and incubated for 30 min with stirring. Excess SMPB was removed using a PD MiniTraP G-25 column. A 2-fold excess of SH-PEG-NH_2_ in PBS was added to the resulting protein solution and incubated for 30 min with stirring. DARPin-LoPE-PEG was filtered from low molecular weight reaction products using a PD MidiTraP G-25 column.

### 4.6. Conjugation of UCNP with DARPin-LoPE-PEG

1-ethyl-3-(3-dimethylaminopropyl) carbodiimide (EDC) up to 2 mM and N-hydroxy sulfo-succinimide (sulfo-NHS) up to 5 mM were added to the UCNP-PMAO suspension in MES buffer (100 mM MES, 150 mM NaCl, pH 6.0). Mixture was incubated for 20 min with stirring, and then the UCNP-PMAO was washed twice with PBS. A solution of DARPin-LoPE-PEG in PBS was added to the suspension and incubated for 2 h with stirring. The obtained complexes UCNP-PMAO-PEG-DARPin-LoPE (UCNP-DARPin-LoPE) were washed three times with PBS to remove unbound protein molecules.

The DARPin-LoPE protein concentration in the complexes was determined by the BCA method using the Pierce™ BCA Protein Assay Kit (Thermo, Rockford, IL, USA) according to the manufacturer’s recommendations. A suspension of UCNP-PEG with the same concentration of UCNP was used as a blank.

To obtain non-targeted UCNP-PEG particles, SH-PEG-NH_2_ was conjugated with UCNP-PMAO, as described above for DARPin-LoPE-PEG.

### 4.7. Specificity of Nanocomplexes Binding to Cell Surface 

Cultures of human breast adenocarcinoma cells SKBR-kat (SKBR-3 cells transfected with TurboFP635 fluorescent protein according to the protocol described in [44], Figure S4) and Chinese hamster ovary cell CHO were used. The presence of the HER2 receptor on the surface of SKBR-kat cells was confirmed by staining them with HER2-specific monoclonal antibodies conjugated to a fluorescent dye (Herceptin-FITC). SKBR-kat and CHO cells were cultured in McCoy’s 5A medium (HyClone Laboratories Inc., Logan, NE, USA) supplemented with L-glutamine and 10% fetal bovine serum (HyClone Laboratories Inc., Logan, NE, USA) at 37 °C and 5% CO_2_. 

For the experiment the cells were seeded onto glass coverslips placed in the wells of a 6-well culture plate at a concentration of 2.5 × 10^5^ cells/mL and grown for 24 h. After that, a suspension of UCNP-PEG/UCNP-DARPin-LoPE was added to the culture medium to a final concentration of 1 μg/mL. The cells were incubated in the presence of nanoparticles/complexes at 4 °C for 1 h. Then, the cells were washed with PBS and fixed with 4% formaldehyde solution for 30 min at 24 °C in the dark. Formaldehyde was removed, and cells were washed 3 times with PBS and 1 time with deionized water. The coverslips were removed from the wells of the plate, dried and placed in a drop of glycerol on glass slides, and then sealed. The binding specificity of the nanoparticles/complexes to the cell surface was investigated by wide-field fluorescence microscopy. The UCNPs’ photoluminescent signal was recorded in the range 420–840 nm with excitation at a wavelength of 980 nm.

The average level of the photoluminescent signal over the selected image areas containing cells was quantified using the ImageJ software Version 1.24o (National Institutes of Health, USA). The significance of differences between groups was tested using the nonparametric Kruskal-Wallis *H*-test using the STATISTIKA program (Dell, Round Rock, TX, USA).

### 4.8. In Vitro Cytotoxicity Assay

The specific cytotoxicity of UCNP-PEG/UCNP-DARPin-LoPE was assessed on SKBR-kat and CHO cultures by MTT assay [47]. SKBR-kat and CHO cells at a concentration of 2 × 10^3^ cells/mL were seeded in a 96-well plate (Corning, Glendale, CA, USA) and cultured for 12 h. The medium was replaced with the growth medium containing the UCNP-PEG/UCNP-DARPin-LoPE at concentrations of 10^−6^–10^3^ μg/mL, and the cells were incubated for 96 h. Then the growth medium was replaced again with a fresh one (100 μL) containing MTT reagent (3-[4,5-dimethylthiazol-2-yl]-2,5-diphenyltetrazolium bromide) (PanEco, Moscow, Russia) at a concentration of 0.5 mg/mL and cells were incubated for 4 h. The medium with MTT was replaced with DMSO (PanEco, Moscow, Russia). The plate was shaken on an OS-20 orbital shaker (Biosan, Latvia) for 5 min until the formazan crystals were completely dissolved. The optical density of the solution in each well was measured using a Synergy MX plate spectrophotometer (BioTek, Winooski, VT, USA) at 570 nm. The data obtained were analyzed using the GraphPad Prism 6.0 software (GraphPad Software). The dose-response curves obtained were used to calculate the half inhibitory concentration (IC_50_) and its 95% confidence interval.

### 4.9. In Vivo Tumor Visualization

The experiments were carried out on immunodeficient mice of the BALB/c Nude line (females, 6–8 weeks old, weight 19–22 g). The animals were purchased from the Animal breeding facility “Pushchino” of the Institute of Bioorganic Chemistry of the RAS. All experimental procedures were approved by the Animal Care and Use Committee of Nizhny Novgorod State University.

The animals were kept in ventilated polypropylene cages at 12 h light regime with access to sterilized food and water *ad libitum*. Mice were subcutaneously inoculated into the thighs with SKBR-kat human breast adenocarcinoma cells in the amount of 8 million/animal. UCNP-PEG/UCNP-DARPin-LoPE were injected intratumorally (at a dose of 10 μg/g of animal weight) when the tumors reached a diameter of ~10 mm.

Fluorescent experimental tumors SKBR-kat, expressing far-red fluorescent protein TurboFP635, were visualized in vivo with setup for surface fluorescence imaging DVS-03 (IPLIT RAS, Russia). Fluorescent images were obtained by using LED with a wavelength of 590 nm and a power of 90 mW, and a bandpass filter 650/50 nm.

Imaging of UCNP-PEG/UCNP-DARPin-LoPE in experimental tumors was carried out in vivo at the whole organism level using a whole-animal imaging setup DVS-02, arranged for anti-stokes luminescence detection. PL images of the animals were obtained in the range of 485–831 nm under excitation at 980 nm with external semiconductor laser module ATC-C4000-200AMF-980-5-F200. 

## 5. Conclusions

A new type of theranostic complexes was obtained based on upconversion nanoparticles coated with polyethylene glycol and functionalized with the recombinant target toxin DARPin-LoPE. The obtained complexes bind to HER2-overexpressing human breast adenocarcinoma cells and demonstrate selective cytotoxicity against this type of cancer cells. The intravital visualization of the UCNP-based complexes proved the ability to use them for photoluminescent diagnosis of cancer, while toxin presence provides therapeutic properties of the created theranostic agents.

## Figures and Tables

**Table 1:** The hydrodynamic diameter and zeta potential of UCNP and nanocomplexes.

	Average Size, nm	PDI	ζ-Potential, mV
UCNP-PMAO	146 ± 20	0.258	−50 ± 3
UCNP-PEG	165 ± 35	0.223	−27 ± 5
UCNP-DARPin-LoPE	179 ± 27	0.221	−18 ± 2

## Supplementary Materials

The following are available online, Figure S1: Diffraction pattern of synthesized nanocrystals α-NaY_0.794_Yb_0.2_Tm_0.006_F_4_ with a cubic crystal lattice and β-NaY_0.794_Yb_0.2_Tm_0.006_F_4_ with a hexagonal crystal lattice, obtained with a wide angle X-ray diffraction (XRD) instrument Rigaku Miniflex600 (Cu, λ = 1.54184 A), Figure S2: Fourier transform infrared spectroscopy (FTIR) spectra of UCNP, PMAO, UCNP-PMAO, DARPin-LoPE and UCNP-DARPin-LoPE, Figure S3: The dependence of the viability of CHO (HER2−) and SKBR-kat (HER2+) cells on the UCNP and DARPin-LoPE concentration, Figure S4: The SKBR-kat cell line creation.
