# Supplementary material for: UCNP-based Photoluminescent Nanomedicines for Targeted Imaging and Theranostics of Cancer
Source: Molecules. 2020 Sep 19;25(18):4302. doi: 10.3390/molecules25184302 (PMC7571190; doi:10.3390/molecules25184302)
Supplement: Supplementary file 1 [file molecules-25-04302-s001.pdf]

Supplementary Material

UCNP-based photoluminescent nanomedicines for targeted imaging and theranostics of cancer

Evgenii L. Guryev[1], Anita S. Smyshlyaeva[1], Natalia Y. Shilyagina[1], Evgeniya A. Sokolova[1], Samah Shanwar[1], Alexey B. Kostyuk [1], Alexander V. Lyubeshkin[2], Alexey A. Schulga[3], Elena V. Konovalova[3], Quan Lin[4], Indrajit Roy[5], Irina V. Balalaeva[1]*, Sergey M. Deyev[3,6], Andrei V. Zvyagin[1,6]

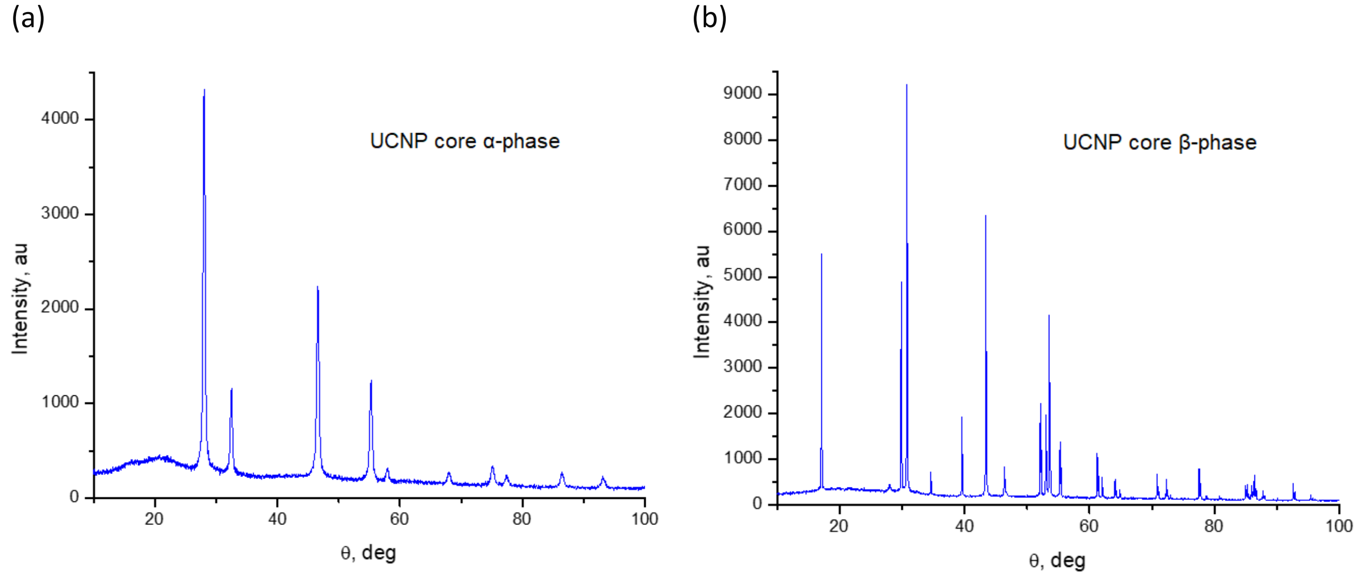

Figure S1. Diffraction pattern of synthesized nanocrystals α-$NaY_{0.794}Yb_{0.2}Tm_{0.006}F_4$ with a cubic crystal lattice (a) and β-$NaY_{0.794}Yb_{0.2}Tm_{0.006}F_4$ with a hexagonal crystal lattice (b), obtained with a wide angle X-ray diffraction (XRD) instrument Rigaku Miniflex600 (Cu, λ = 1.54184 A). θ - angle of incidence of the x-ray on the sample. The β-phase is characterized by the presence of a signal at 17 degrees.

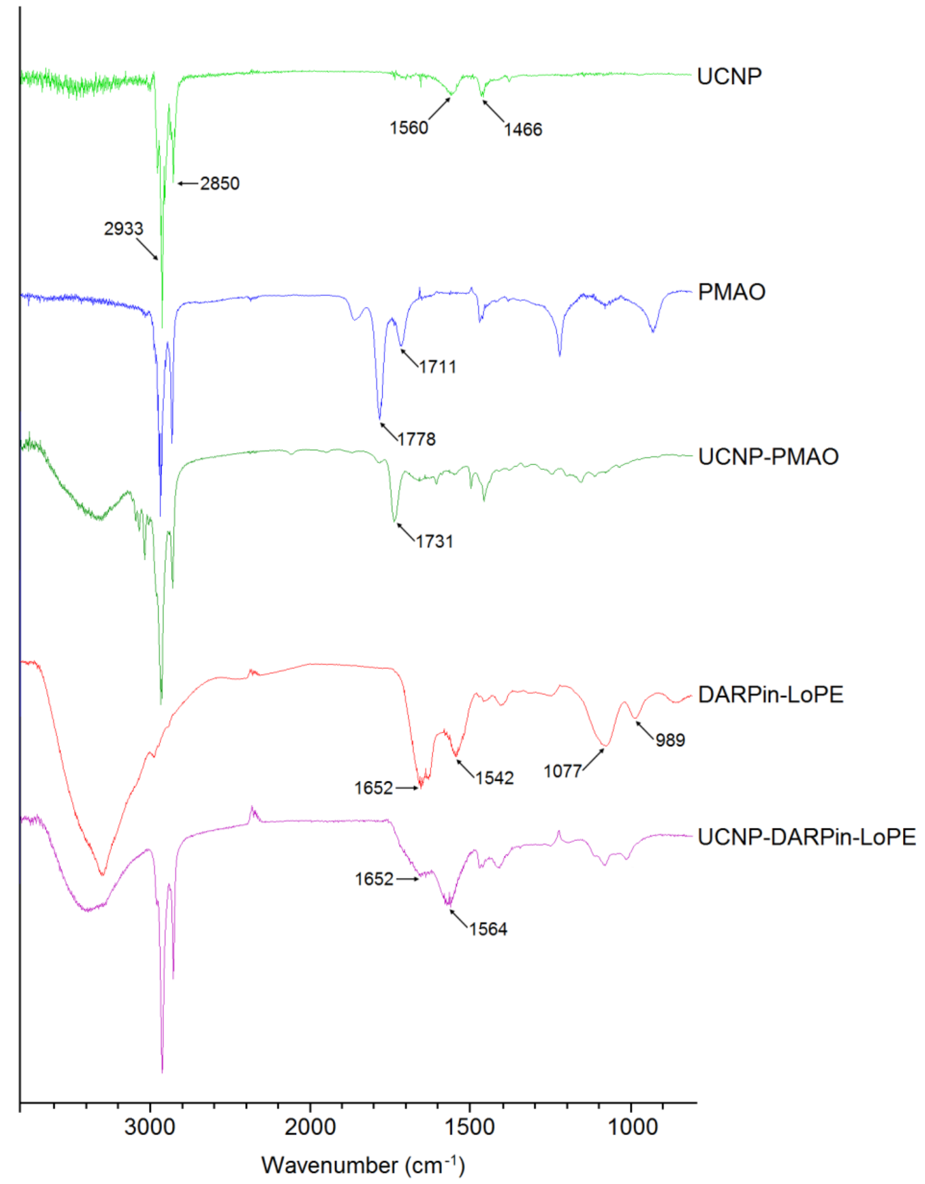

Figure S2. Fourier transform infrared spectroscopy (FTIR) spectra of UCNP, PMAO, UCNP-PMAO, DARPin-LoPE and UCNP-DARPin-LoPE. FTIR spectra were recorded on the Shimadzu IR Prestige-21 spectrometer. The 2933 cm^{-1} and 2850 cm^{-1} bands are assigned to the asymmetric stretch and symmetric stretch of $-CH_2$ groups of oleic acid (covering as-synthesized UCNP) and PMAO, and found in all materials except DARPin-LoPE. In the case of UCNP fluctuations on 1560 cm^{-1} и 1466 $см^{-1}$ were recorded, that correspond to asymmetric stretch and symmetric stretch vibrations of COO^{-} group of oleic acid. A sample of pure PMAO showed a band 1778 cm^{-1} corresponding to the vibrations of anhydride. Also, due to the hydrolysis of PMAO, we observed a band at 1711 cm^{-1}, which corresponds to the formed -COOH group. The UCNP-PMAO sample showed a shift in the band corresponding to anhydride vibrations up to 1731 cm^{-1}. For pure DARPin-LoPE, a spectrum with bands 1652 cm^{-1} (amide I band), 1542 cm^{-1} (amide II band) was obtained, as well as bands at 1077 cm^{-1} and 989 cm^{-1}, which correspond to P-O vibrations (from PBS buffer components). The UCNP-DARPin-LoPE nanocomplexes showed a characteristic band of 1652 cm^{-1} (amide I band) corresponding to DARPin-LoPE oscillations. The amide II band для UCNP-DARPin-LoPE did not occur in well-resolved shape. Besides we observed the strong vibration at 1564 cm^{-1}, which probably correlates with new amide bonds formed as a result of DARPin-LoPE attachment.

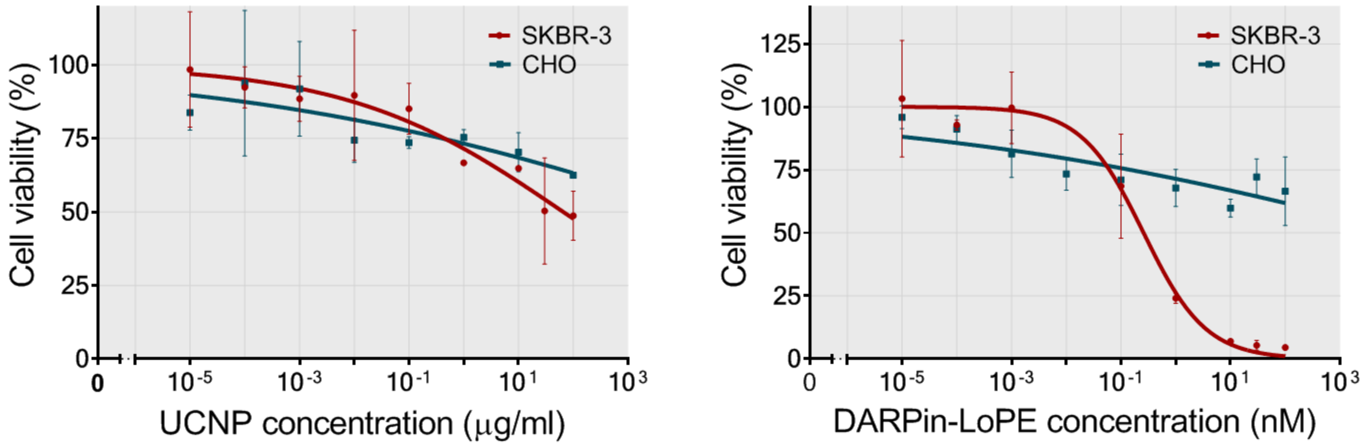

Figure S3. The dependence of the viability of CHO (HER2–) and SKBR-kat (HER2+) cells on the UCNP (left) and DARPin-LoPE (right) concentration. Incubation time 96 hours.

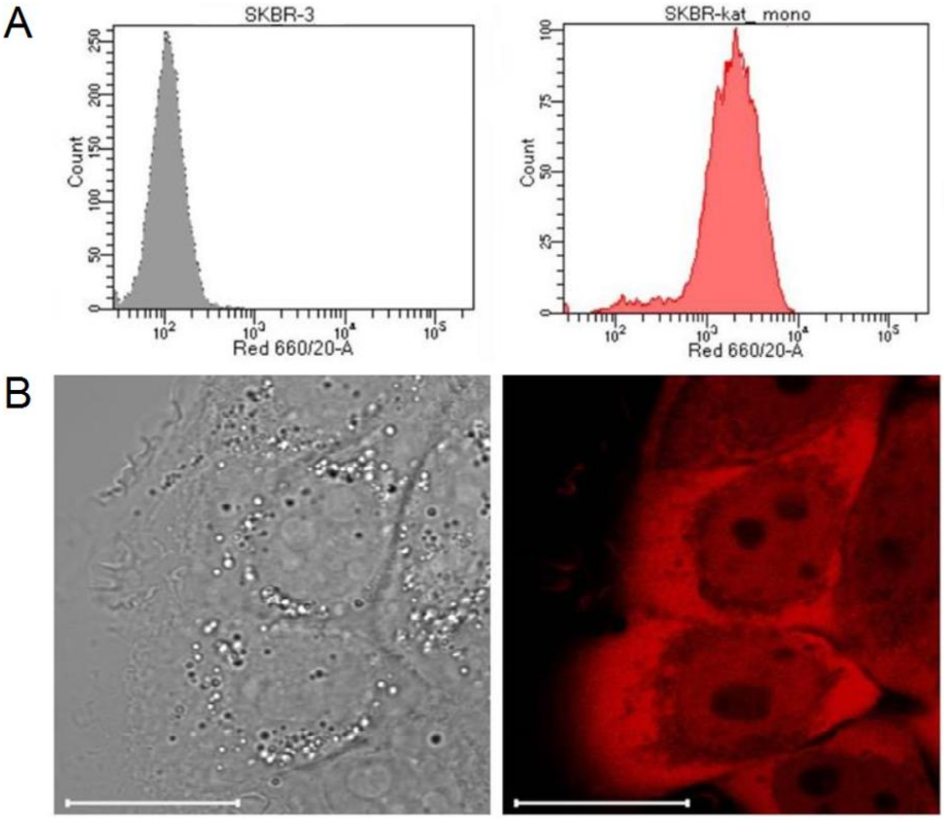

Figure S4. The SKBR-kat cell line creation. (A) Flow cytometry analysis of cell distribution according to fluorescence signal (λex 633 nm, λem 650-670 nm). Cells of the parental cell line SKBR-3 (left) and SKBR-kat (right). (B) Confocal images of SKBR-kat cells in transmitted light (left) and in red fluorescence channel, λex 633 nm, λem 650-670 nm (right). Bar, 20 µm.

SKBR-3 parental cell line (ATCC® HTB-77™) was stably transfected with gene of far-red fluorescent protein TurboFP635, known also as Katushka, by lipofection technique. Cells were seeded in 24-well plate, grown overnight and then treated by complex of plasmid vector pTurboFP635 (Evrogen, Russia) with Lipofectamin 3000 (Invitrogen, USA) according to the manufacturer's protocols. After 24 h incubation the medium was exchanged to fresh full growth medium, and the cells were cultured for 48 h. To select transfected cells, they were then cultured in media with increasing concentrations of G418 (Gibco, USA), up to 2 µg/ml. Cells selected on antibiotic containing medium were then sorted using FACS Aria III cell sorter (BD, США); the cell with higher red fluorescence level (λex 561 nm, λem 600-620 nm) were collected for further expansion. The sort/expansions cycle was repeated for three types to obtain the brightest and stably transfected fluorescent cell population. At the last stage, the single cell sort was performed and the monoclonal line with bright fluorescence, morphological and growth parameters close to parental cell line, and high level of HER2 expression was chosen for further experiments and established as *SKBR-kat.*
